# Vascular endothelial growth factor-A and chemokine ligand (CCL2) genes are upregulated in peripheral blood mononuclear cells in Indian amyotrophic lateral sclerosis patients

**DOI:** 10.1186/1742-2094-8-114

**Published:** 2011-09-09

**Authors:** Pawan K Gupta, Sudesh Prabhakar, Chandrika Abburi, Neel K Sharma, Akshay Anand

**Affiliations:** 1 Department of Neurology, Post Graduate Institute of Medical Education and Research (PGIMER), Chandigarh-160012, India

## Abstract

**Background:**

We have earlier shown that protein levels of vascular endothelial growth factor-A (VEGF-A) and chemokine ligand-2 (CCL2) were elevated in Indian amyotrophic lateral sclerosis (ALS) patients. Here, we report the mRNA levels of VEGF-A and CCL2 in Indian ALS patients since they display extended survival after disease onset.

**Methods:**

VEGF-A and CCL2 mRNA levels were measured in peripheral blood mononuclear cells (PBMCs) of 50 sporadic Indian ALS patients using Real Time Polymerase Chain Reaction (PCR) and compared with normal controls (n = 50). Their levels were adjusted for possible confounders like cigarette smoking, alcohol and meat consumption.

**Results:**

VEGF-A and CCL2 mRNA levels were found to be significantly elevated in PBMCs in ALS patients as compared to controls. PBMCs from definite ALS revealed higher VEGF-A mRNA expression as compared to probable and possible ALS. CCL2 mRNA levels were found to be unaltered when definite, probable and possible ALS were compared. PBMCs from patients with respiratory dysfunction showed much higher VEGF-A and CCL2 elevation when compared to patients without respiratory dysfunction. No association of smoking, alcohol and meat consumption with VEGF-A and CCL2 was observed after analyzing the data with univariate and multivariate analysis.

**Conclusion:**

VEGF-A and CCL2 mRNA upregulation in PBMCs may have a clinico-pathological/etiological/epidemiological association with ALS pathogenesis. The cross-cultural and cross-ethnic investigations of these molecules could determine if they have any role in enhancing the mean survival time unique to Indian ALS patients.

## Introduction

Amyotrophic lateral sclerosis (ALS) is a neurodegenerative disorder characterized by selective loss of motor neuron. Vascular endothelial growth factor-A (VEGF-A) is a dimeric secreted polypeptide that was discovered first in the VEGF family which also includes placental growth factor (PLGF), VEGF-B, VEGF-C, VEGF-D and VEGF-E. VEGF-A stimulates growth of blood vessels during embryonic development and helps in proliferation of blood collaterals in diseased conditions including ALS through a tyrosine kinase dependent VEGF receptor-2 (VEGFR2) [1]. Apart from angiogenesis, VEGF-A is suggested to exert direct neuroprotection via VEGFR2 and neuropilin-1 (NP-1) in animal models and patients of various neurodegenerative disorders [2]. Mice having homozygous deletion in hypoxia response element (HRE) of VEGF-A promoter (VEGF^δ/δ^) were reported to develop symptoms like classical ALS [3] and conversely, intrathecal transplantation of stem cells overexpressing VEGF-A delays the onset and progression of ALS in superoxide dismutase-1 (SOD1) mutated transgenic mouse by downregulating proapoptotic proteins and activating phosphatidylinositol 3-kinase/protein kinase B (PI3-K/Akt) anti apoptotic pathway [4]. On the other hand, chemokine ligand-2 (CCL2), a proinflammatory molecule, may impart neuroprotection in ALS against glutamate induced excitotoxicity either by reducing release of glutamate and/or increasing efficiency of astrocytes to clear glutamate at synapses [5].

Indian ALS patients are known to exhibit significantly extended survival duration after disease onset as compared to Western ALS patients [6-8]. We recently reported that augmented biofluids VEGF-A and CCL2 protein may be associated with increased survival duration of Indian ALS patients [9]. We now measured the mRNA expression of VEGF-A and CCL2 in peripheral blood mononuclear cells (PBMCs) of these patients.

## Subjects and methods

50 patients, born in North India and diagnosed with ALS were included from a convenience sample of Neurology outpatient, post graduate institute of medical education and research (PGIMER), Chandigarh after obtaining informed consent as a part of research protocol as per institute ethical committee guidelines (No. 7055-PG-1Tg-05/4348-50). Based on the "El Escorial criteria", there were 25 definite ALS patients, 15 individuals were probable ALS and remaining 10 were possible ALS at the time of sample collection. ALS-functional rating score-revised (ALSFRS-R) revealed that 11 patients had respiratory dysfunction such as orthopnea and dyspnea accompanied with other respiratory insufficiencies, although none of the patients needed respiratory support [10]. ALS patients with history of diabetic neuropathy, glaucoma, pre-eclampsia, stroke, those receiving riluzole, anti inflammatory drugs, antioxidants or other treatment were excluded. 50 genetically unrelated healthy normal controls without any apparent health problems such as hypertension, diabetes, heart disease etc were included for comparison. The subjects were categorized as cigarette smokers and never smokers, alcohol consumers and nonalcoholics, vegetarian and non-vegetarian (or meat consumers) using a standard questionnaire as per published criteria [11]. The clinical and demographic details of subjects published earlier [9] have also been reproduced here in Table 1.

**Table 1:** Characteristics of the subjects

Subjects	**Age (y)**^**†**^	M/F (n)	Age of onset (y)	Disease duration^‡ ^(mo)	B/L (n)	Smokers (n)	Alcohol consumers (n)	Non-vegetarian (n)
ALS	47.4 ± 12.4	38/12	46.2 ± 12.8	19.0 ± 12.7	8/42	12	12	20

Controls	40.0 ± 12.8	39/11				10	14	27

Clinical and demographic details of subjects. ALS, amyotrophic lateral sclerosis; n, Number; M, male; F, female; y, years; mo, months; B, bulbar; L, limb; Age, age of onset, duration of disease are indicated as mean ± standard deviation (SD). † Unpaired, independent 2-tailed student *t *test analysis showed that mean age differ significantly among the groups (p = 0.004). ‡ Duration of disease is the interval between appearance of first symptom of ALS and collection of sample. ALS subjects were asked to provide all clinical and demographic details at the age of disease-onset.

PBMCs were isolated as per Histopaque-1077 (Sigma, USA) datasheet. Briefly, 6.0 ml blood was collected from each subject and layered on equal volume of Histopaque-1077. It was then centrifuged at 1800 rpm for 30.0 mins at room temperature and PBMCs were collected from plasma/Histopaque-1077 interface and preserved in RNA later (Sigma, USA) at -80°C until used.

Total RNA was extracted from PBMCs using RNAeasy columns (Qiagen, USA). RNA concentration was measured by taking absorbance at 260.0 nm. About 500.0 ng - 5000.0 ng total RNA was used to synthesize cDNA according to RevertAid™ first strand cDNA kit (Fermentas, USA).

Real Time Polymerase Chain Reaction (PCR) was used to quantitate expression of VEGF-A and CCL2 mRNA using published primers [12-14]. Methodology of Real Time PCR has been elaborated in "Additional File 1".

Because the data was normally distributed as indicated by quintile-quintile (Q-Q) plot, unpaired, independent, 2-tailed student *t *test and one-way analysis of variance (ANOVA) followed by Fisher's least significant difference (LSD) *post hoc *analysis was applied for statistical comparisons. Crude and adjusted odds ratio (OR) was evaluated by univariate and multivariate logistic regression respectively to check any possible influence of smoking, alcohol and meat consumption on VEGF-A and CCL2 mRNA levels and χ^2 ^(chi square) test was performed to find significance level.

*p-*value was considered significant at ≤0.05. Statistical analysis was performed by statistical package and service solutions (SPSS) 16 software. Results were analyzed by two independent and masked researchers.

## Results

**Figure 1:**
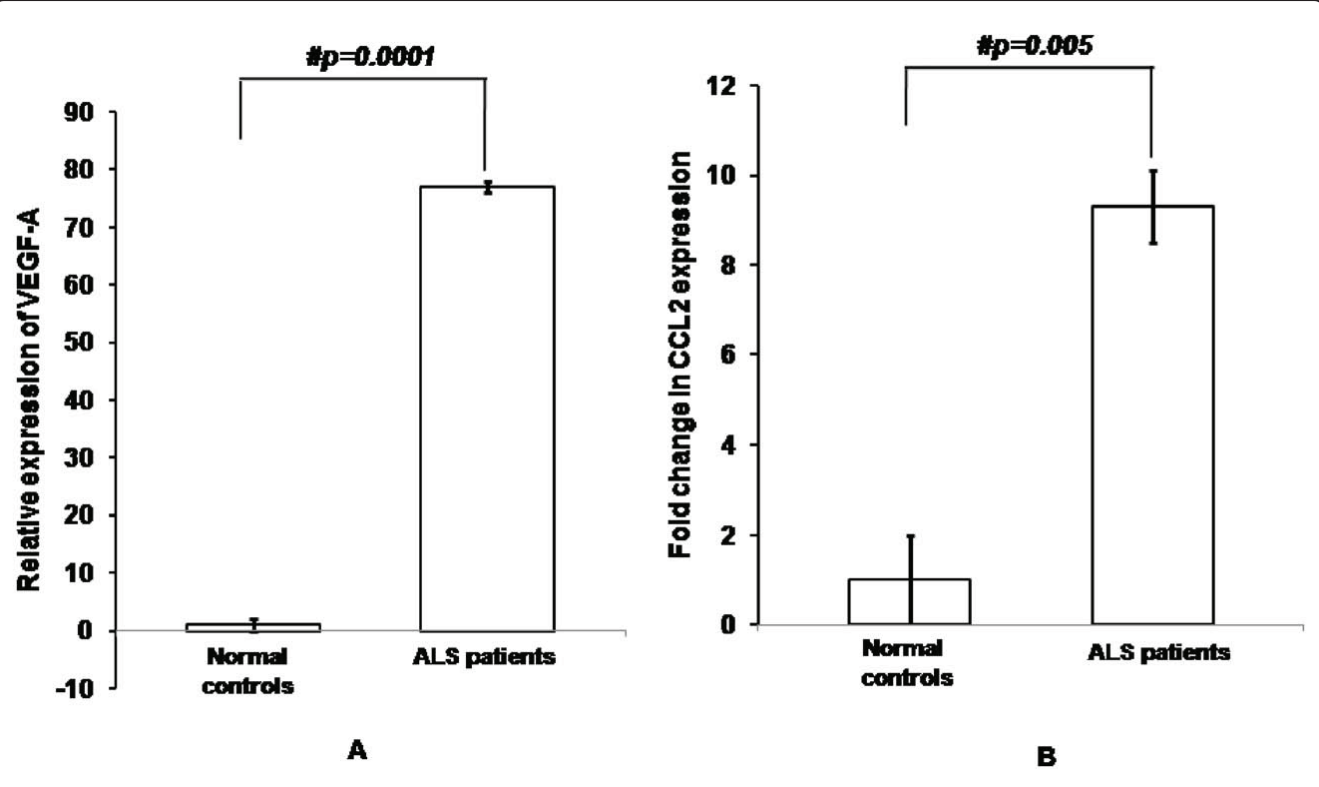
**Relative mRNA expression of VEGF-A (A) and CCL2 (B) in PBMCs of ALS patients**. Values are plotted as mean ± SE (Standard error) in the bar diagram. Data was analyzed by unpaired, independent 2-tailed student *t *test. # indicates significant difference among the groups (p < 0.05). Expression of VEGF-A and CCL2 were normalized to expression of endogenous control b-actin. ALS, amyotrophic lateral sclerosis; VEGF-A, vascular endothelial growth factor-A; CCL2, chemokine ligand-2; PBMCs, peripheral blood mononuclear cells.

**Figure 2:**
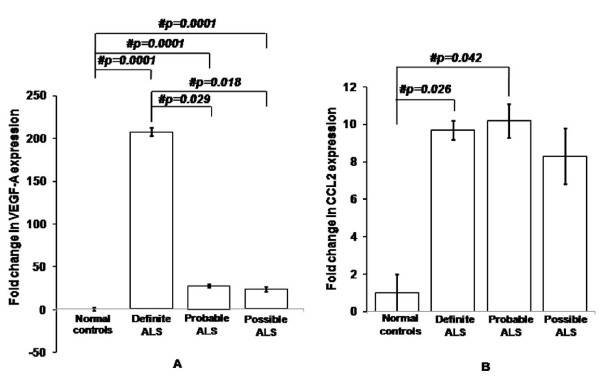
**Relative mRNA expression of VEGF-A (A) and CCL2 (B) in PBMCs of definite, probable and possible ALS patients**. Values are plotted as mean ± SE (Standard error) in the bar diagram. Data was analyzed by one-way analysis of variance (ANOVA) followed by Fisher's least significant difference (LSD) *post hoc *test. # indicates significant difference among the groups (p < 0.05). Expression of VEGF-A and CCL2 were normalized to expression of endogenous control b-actin. Basal VEGF-A mRNA expression in normal control group is represented by x-axis in part "A". ALS, amyotrophic lateral sclerosis; VEGF-A, vascular endothelial growth factor-A; CCL2, chemokine ligand-2; PBMCs, peripheral blood mononuclear cells.

Real Time PCR indicates that VEGF-A expression is 77-fold higher in ALS than controls (Figure 1A; p = 0.0001). CCL2 mRNA has shown an increment of 9.5-fold in ALS than controls (Figure 1B; p = 0.005). There was elevated VEGF-A mRNA expression in definite ALS patients in comparison to controls, probable and possible ALS (Figure 2A; p = 0.0001, p = 0.029 and p = 0.018 respectively). Further, both probable and possible ALS patients were shown to have higher VEGF-A than controls (Figure 2A; p = 0.0001 and p = 0.0001 respectively). However, CCL2 levels did not vary between definite, probable and possible ALS cases (Figure 2B; p > 0.05).

**Figure 3:**
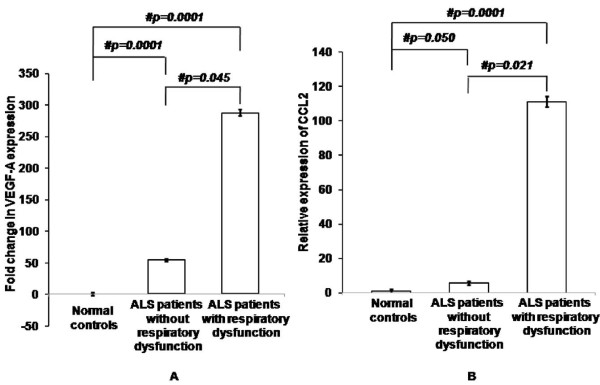
**Fold change in expression of VEGF-A (A) and CCL2 (B) in PBMCs of ALS patients with respiratory dysfunction**. Values are plotted as mean ± SE (Standard error) in the bar diagram. Data was analyzed by one-way analysis of variance (ANOVA) followed by Fisher's least significant difference (LSD) *post hoc *test. # indicates significant difference among the groups (p < 0.05). Expression of VEGF-A and CCL2 were normalized to expression of endogenous control b-actin. Basal VEGF-A mRNA expression in normal control group is represented by x-axis in part "A". ALS, amyotrophic lateral sclerosis; VEGF-A, vascular endothelial growth factor-A; CCL2, chemokine ligand-2; PBMCs, peripheral blood mononuclear cells.

To find association of respiratory dysfunction, VEGF-A and CCL mRNA levels were reanalyzed among ALS patients with respiratory dysfunction and those without respiratory dysfunction. Significantly increased VEGF-A and CCL2 was observed in ALS patients with respiratory dysfunction as compared to patients without respiratory dysfunction (Figure 3A-B; p = 0.045 and p = 0.021 respectively)

No association of cigarette smoking, alcohol and meat consumption with VEGF-A (Table 2) and CCL2 (data not shown) mRNA was observed upon univariate and multivariate analysis.

**Table 2:** Crude and adjusted OR for VEGF-A mRNA

	**OR (95% CI)**^**†**^	p*	**Adj. OR (95% CI)**^**‡**^	p*
**VEGF-A mRNA**				
Smoking	0.8 (0.2-4.3)	0.8	1.1 (0.2-6.3)	0.8
Alcohol consumption	1.0 (0.2-4.3)	0.9	0.9 (0.2-4.5)	0.9
Meat consumption	0.8 (0.2-3.0)	0.8	0.8 (0.2-3.3)	0.8

Never smoking/	1.0		1.0	
Nonalcoholic/				
Vegetarian**				

† Univariate logistic regression was used to calculate crude OR. ‡ Multivariate logistic regression was used to adjust the effect of smoking on VEGF-A mRNA levels with alcohol and meat consumption as covariates. Likewise, effect of alcohol and meat consumption on VEGF-A is also adjusted for covariates. *χ^2 ^(chi square test) was used to test the level of significance. ** Never smoking, nonalcoholic and vegetarian diet is considered as reference group. VEGF-A, vascular endothelial growth factor-A; OR, odds ratio; CI, confidence interval; Adj, adjusted.

## Discussion

It has been reported that median survival duration of Indian ALS patients is ~9 years after disease onset which is significantly higher as compared to their Western counterparts who survive for 3-6 years after disease onset [6-8]. Because of this contradicting presentation, we investigated the levels of VEGF-A and CCL2 among the Indian ALS patients.

The increased PBMCs VEGF-A and CCL2 expression in our patients may suggest the pathophysiological involvement of circulating monocytes and lymphocytes in ALS. The elevated PBMCs VEGF-A is in contrast to previous reports where a profound downregulation of VEGF-A mRNA in SOD1G93A ALS mouse and significantly reduced serum and cerebrospinal fluid (CSF) VEGF-A in ALS patients was observed possibly because of genetic changes in promoter regions [15-17]. Increased serum and CSF VEGF-A reported earlier in ALS and in its different clinical subtype with limb onset and extended disease duration are in agreement with current results [18,19]. However, some studies have failed to detect significant change in serum, plasma and CSF VEGF-A in ALS patients [20,21]. It is believed that the variable study designs including different molecular tools, study power, diverse clinical and genetic spectrum of ALS patients may account for conflicting VEGF-A levels. The increased PBMCs CCL2 is consistent with reports where elevated CCL2 mRNA was observed in spinal cord and skeletal muscles of ALS patient's autopsies and SOD1 mutated ALS mice [14,22].

As VEGF-A and CCL2 are neurotrophic, Indian ALS patients may enhance VEGF-A and CCL2 expression in an attempt to ameliorate excitotoxicity through upregulation of glutamate receptor as reported earlier [5,23]. Increased VEGF-A and CCL2 may promote migration and differentiation of VEGF receptor 1 (VEGFR1), VEGFR2 and chemokine receptor 2 (CCR2) expressing adult neural progenitor cell into neuronal and glial phenotypes at the site of injury [24,25]. Whether their upregulation represent any compensatory response towards extended survival of Indian ALS patients should be evaluated in future comparable cross-cultural and cross-ethnic ALS population where survival is longer. It must be emphasized that mean survival duration of reported ALS patients could not be ascertained.

Since elevated CCL2 initiates inflammatory reaction by increasing production of nitric oxide and other inflammatory chemokines from unregulated monocytes/macrophages [26] and VEGF-A is known to recruit leukocytes at the site of brain injury by increasing vascular permeability [27], it is possible that the high VEGF-A and CCL2 in our ALS patients may exert limited inflammatory responses associated with neuroprotection [28].

At this moment, we are not able to state whether the increased VEGF-A and CCL2 mRNA is a consequence of genetic and/or epigenetic changes of upstream regulatory sequences, altered transcriptional regulation or amyotrophy and thus the present report lays the foundation for future studies to screen promoter elements of VEGF-A and CCL2 in Indian ALS population for subtle genetic differences. The stress conditions, like respiratory problems, may also modify transcriptional gene regulation as indicated by increased VEGF-A and CCL2 mRNA expression in the 11 ALS patients with respiratory dysfunction and signifies a possible association with hypoxia (Figure 3).

Based on existing literature [29,30], elevated VEGF-A and CCL2 in definite ALS may represent the possibility of relatively extensive extra central nervous system (CNS) involvement and higher degree of nerve endings arborization at neuromuscular junction than probable and possible ALS, however, neuroanatomical architecture of neuromuscular junction has not been evaluated. The possibility of increased VEGF-A and CCL2, in definite ALS due to respiratory dysfunction, may not be ruled out even though only 28% of all definite ALS cases presented with respiratory symptoms.

## Conclusion

Although it can not be concluded that increased VEGF-A and CCL2 expression contributes towards enhanced survival yet the importance of clinico-pathological, etiological and epidemiological association of increased VEGF-A and CCL2 with survival of Indian ALS patients may not be underestimated and needs further investigations.

## Abbreviations

ALS: amyotrophic lateral sclerosis; ALSFRS-R: ALS functional rating score-revised; ANOVA: analysis of variance; CCL2: chemokine ligand-1; CCR2: chemokine receptor-2; CNS: central nervous system; CSF: cerebrospinal fluid; EDTA: ethylene diamine tetraacetate; HRE: hypoxia response element; LSD: least significant difference; mRNA: messenger ribonucleic acid; NMDA: N-Methyl-D-aspartate; NP-1: neuropilin-1; OR: odds ratio; PBMCs: peripheral blood mononuclear cells; PCR: polymerase chain reaction; PI3-K: phosphatidylinositol 3-kinases; SOD1: superoxide dismutase 1; VEGF: vascular endothelial growth factor; VEGFR1: VEGF receptor-1; VEGFR2: VEGF receptor-2.

## Ethical approval

Ethical approval was obtained by institute ethical committee, PGIMER, Chandigarh, India - 160012 (No. 7055-PG-1Tg-05/4348-50).

## Competing interests

The authors declare that they have no competing interests.

## Authors' contributions

PKG Acquisition of data and writing of manuscript. SP inclusion of patients, grant PI and clinical scoring. CA Acquisition of data. NKS Statistical analysis. AA Interpretation and analysis of data, grant co PI and writing and editing of manuscript. All authors read and approved the final manuscript.

## Supplementary Material

Additional file 1 **Real Time Polymerase Chain reaction (PCR)**. Methodology of Real Time PCR; PCR cycling conditions and amplicon size of VEGF-A and CCL2; sequences and references of primers used. Click here for file
