# Supplementary material for: Vascular endothelial growth factor-A and chemokine ligand (CCL2) genes are upregulated in peripheral blood mononuclear cells in Indian amyotrophic lateral sclerosis patients
Source: J Neuroinflammation. 2011 Sep 9;8:114. doi: 10.1186/1742-2094-8-114 (PMC3177897; doi:10.1186/1742-2094-8-114)
Supplement: Additional file 1 — Real Time Polymerase Chain reaction (PCR). Methodology of Real Time PCR; PCR cycling conditions and amplicon size of VEGF-A and CCL2; sequences and references of primers used. [file 1742-2094-8-114-S1.PDF]

Real Time Polymerase Chain Reaction (PCR):

Real Time PCR was performed in the 48 wells version of Step One™ (Applied Biosystems, USA) using published primers [see table below] with SYBR green Real Time PCR kit (Invitrogen, USA). Each reaction was conducted for 20.0µl containing 10.0µl of 2X SYBR green, 0.20µl ROX dye as passive reference, 0.40µl Taq DNA Polymerase, 0.20µl (200.0nM) of each sense and antisense primers, 50.0ng cDNA template and molecular biology grade water was added to make the volume 20.0µl. The cycling conditions consisted of initial denaturation step for 10.0mins at 95^0C followed by 40 cycles of denaturation at 95^0C for 1.0 min, annealing at 60^0C for VEGF-A and 55^0C for CCL2 for 1.0min and extension at 72^0C for 1.0min. Relative expression was analyzed in ALS subjects using $2^{-\Delta\Delta Ct}$ or comparative Ct (threshold cycle) method after normalization with endogenous control β-actin [see table below] followed by normalization with control group and fluorescence data was obtained at the annealing step. Briefly, the $2^{-\Delta\Delta Ct}$ or comparative Ct method compared Ct values of ALS patients and normal controls whose steps are listed below:

1. Normalization of Ct values of target gene (VEGF-A and CCL2) to Ct of an endogenous control β-actin for both ALS and normal control samples.

   ΔCt (ALS) = Ct (target gene) – Ct (endogenous control β-actin)

   ΔCt (normal control) = Ct (target gene) – Ct (endogenous control β-actin)

2. Normalization of ΔCt (ALS) to ΔCt (normal control) to obtain $\Delta\Delta$Ct.

   $\Delta\Delta$Ct = ΔCt (ALS) – ΔCt (normal control).

3. Fold change expression of target gene (VEGF-A and CCL2) in ALS group

   $X_{ALS}/X_{normal\ control} = 2^{-\Delta\Delta Ct}$

Table Sequences of Real Time PCR Primers

Genes	Sequence (5' → 3')	Amplicon size	Reference (In the main document)
VEGF-A	F: CGA AGT GGT GAA GTT CAT GGA TG	$VEGF_{121}$:403	[12]
	R: TTC TGT ATC AGT CTT TCC TGG TGA G	$VEGF_{165}$:535	
		$VEGF_{189}$:607	
CCL2	F: TCT CGC CTC CAG CAT GAA A	267	[13]
	R: TCC TGA ACC CAC TTC TGC TTG		
β-actin	F: GTG GGG CGC CCC AGG CAC CA	539	[14]
	R: CTC CTT AAT GTC ACG CAC GAT TTC		
